# Supplementary material for: High-Light-Induced Degradation of Photosystem II Subunits’ Involvement in the Albino Phenotype in Tea Plants
Source: Int J Mol Sci. 2022 Jul 31;23(15):8522. doi: 10.3390/ijms23158522 (PMC9369412; doi:10.3390/ijms23158522)
Supplement: Supplementary file 1 [file ijms-23-08522-s001.zip › ijms-1817889-supplementary.pdf]

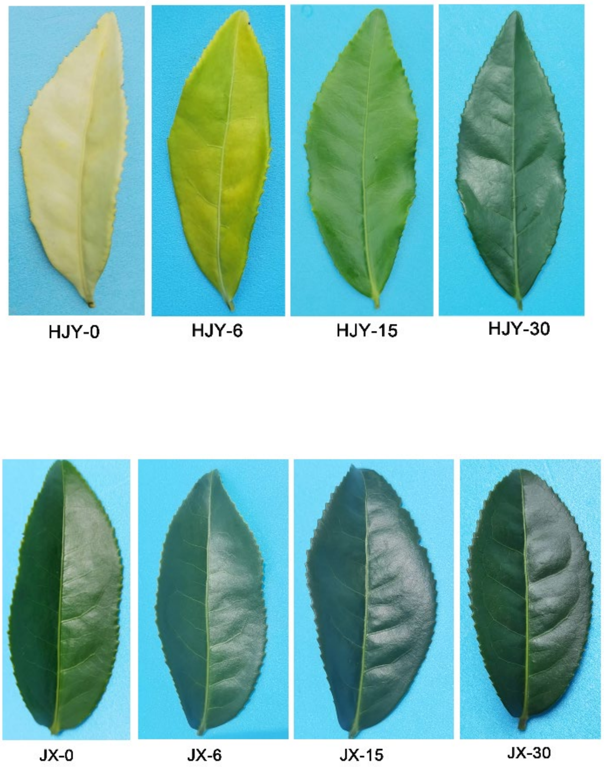

Figure S1. Greening of albino leaf during shading treatment.

HJY, albino cultivar 'Huangjinya'; JX, normal green leaf cultivar 'Jinxuan'; 0, before shading treatment in which the plants grown under high light conditions (diurnal sunlight 1200-1900 $\mu mol/m^2 \cdot s$); 6, 15, 30, the number of days under shading treatment.

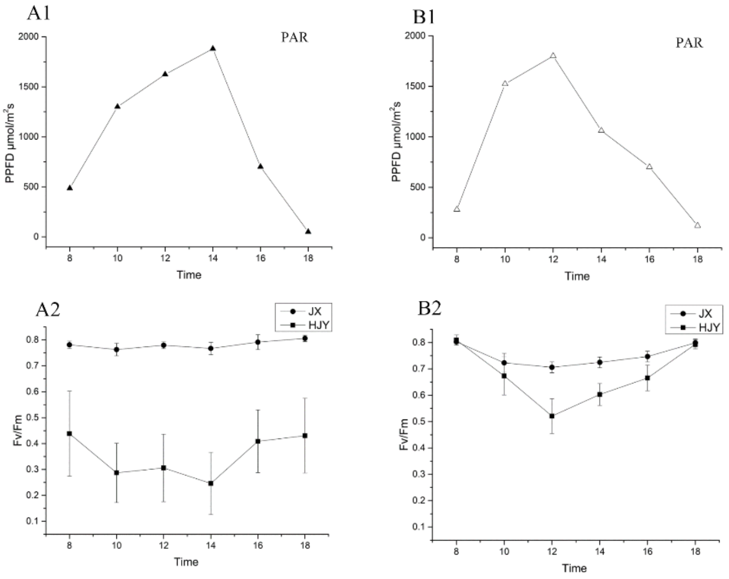

Figure S2. Diurnal changes in Fv/Fm and PAR.

The Fv/Fm was tested on the 4th leaf beneath apex bud and 10 leaves were tested each treatment. HJY, cultivar 'Huangjinya'; JX, cultivar 'Jinxuan'; PPFD, photosynthetic photon flux density; PAR, photosynthetically active radiation; A1, diurnal changes in PAR on the day starting artificial shading treatment; A2, diurnal changes in Fv/Fm of albino leaves of 'HJY' and green leaves of 'JX' on the day starting artificial shading treatment; B1, diurnal changes in PAR on the day ending artificial shading treatment; B2, diurnal changes in Fv/Fm of green leaves of 'HJY' and 'JX' on the day ending artificial shading treatment. The Fv/Fm is presented as mean ± standard deviation of 10 replicated experiments (n=10).

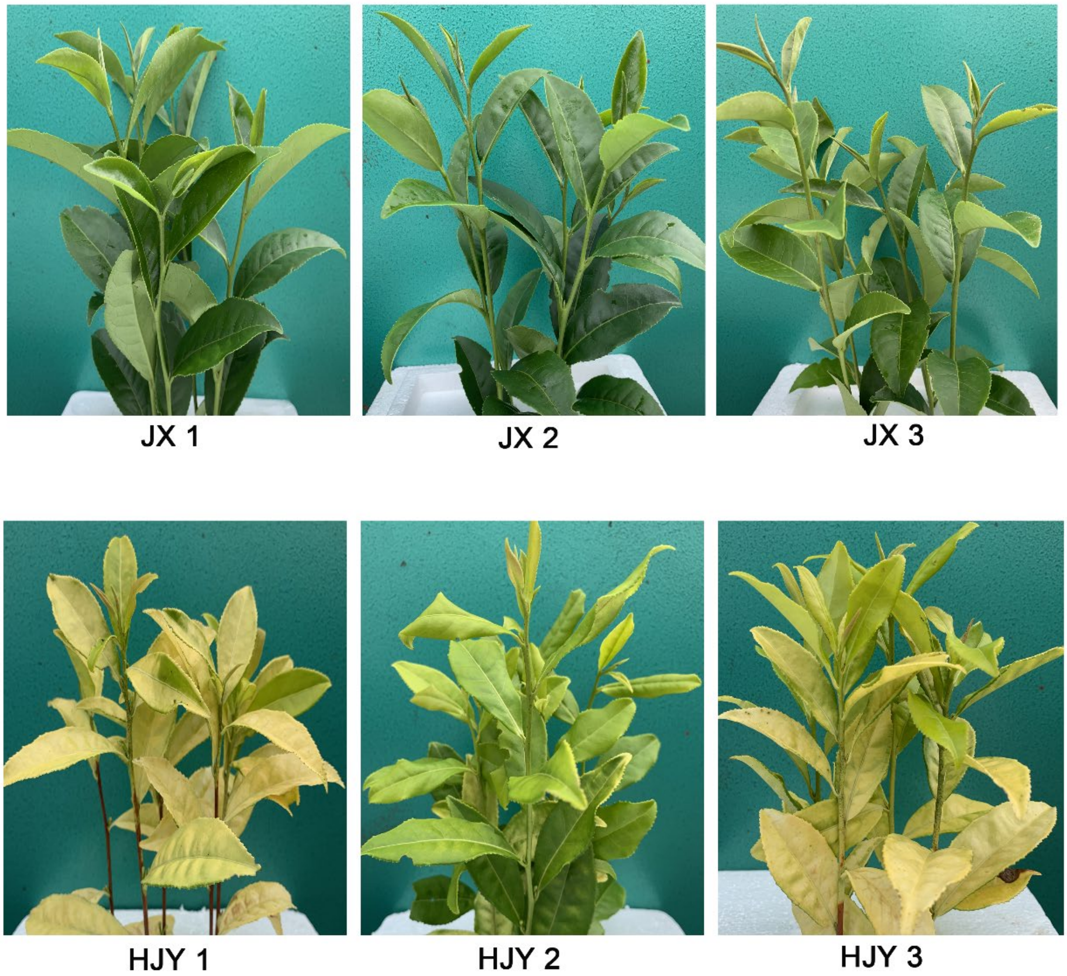

Figure S3. Effect of lincomycin on re-greening of albino shoots under LL condition.

Tea shoots grown in natural sunshine (~1500 μmol/m^2·s) were cut and then incubated in 1 mM lincomycin solution by hydroponics under LL（low light）conditions (400 μmol/m^2·s, 12 hour light and 12 hour dark) for 15 days. HJY, cultivar 'Huangjinya'; JX, cultivar 'Jinxuan'. JX 1, 'JX' before incubated in lincomycin solution; JX 2, 'JX' incubated in water under LL condition for 15 days; JX 3, 'JX' incubated in lincomycin solution under LL conditions for 15 days; HJY 1, 'HJY' before incubated in lincomycin solution; HJY 2, 'HJY' incubated in water under LL condition for 15 days; HJY 3, 'HJY' incubated in lincomycin solution under LL conditions for 15 days.

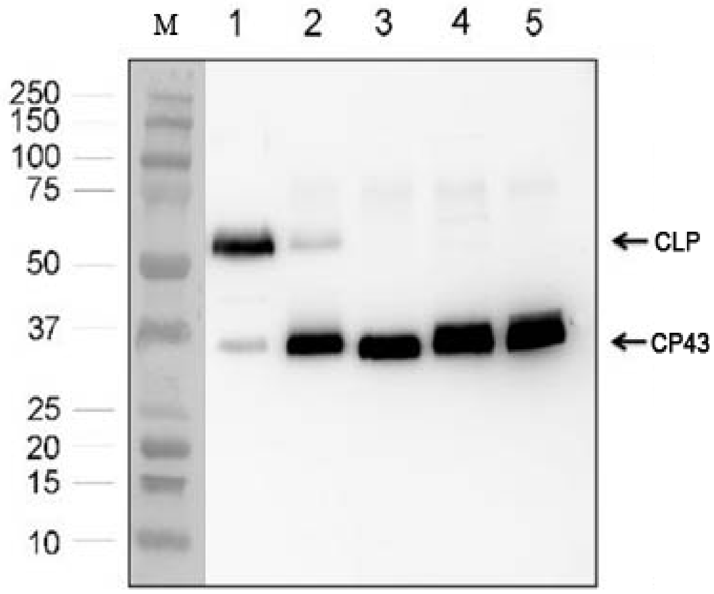

Figure S4. Immunoblotting of CP43 from various plant species.

M, marker; 1, HJY grown under high light condition; 2, HJY under low light condition; 3, *Arabidopsis thaliana* grown under green house condition; 4, *Spinacia oleracea* grown under green house condition; 5, *Solanum lycopersicum* grown under green house condition.

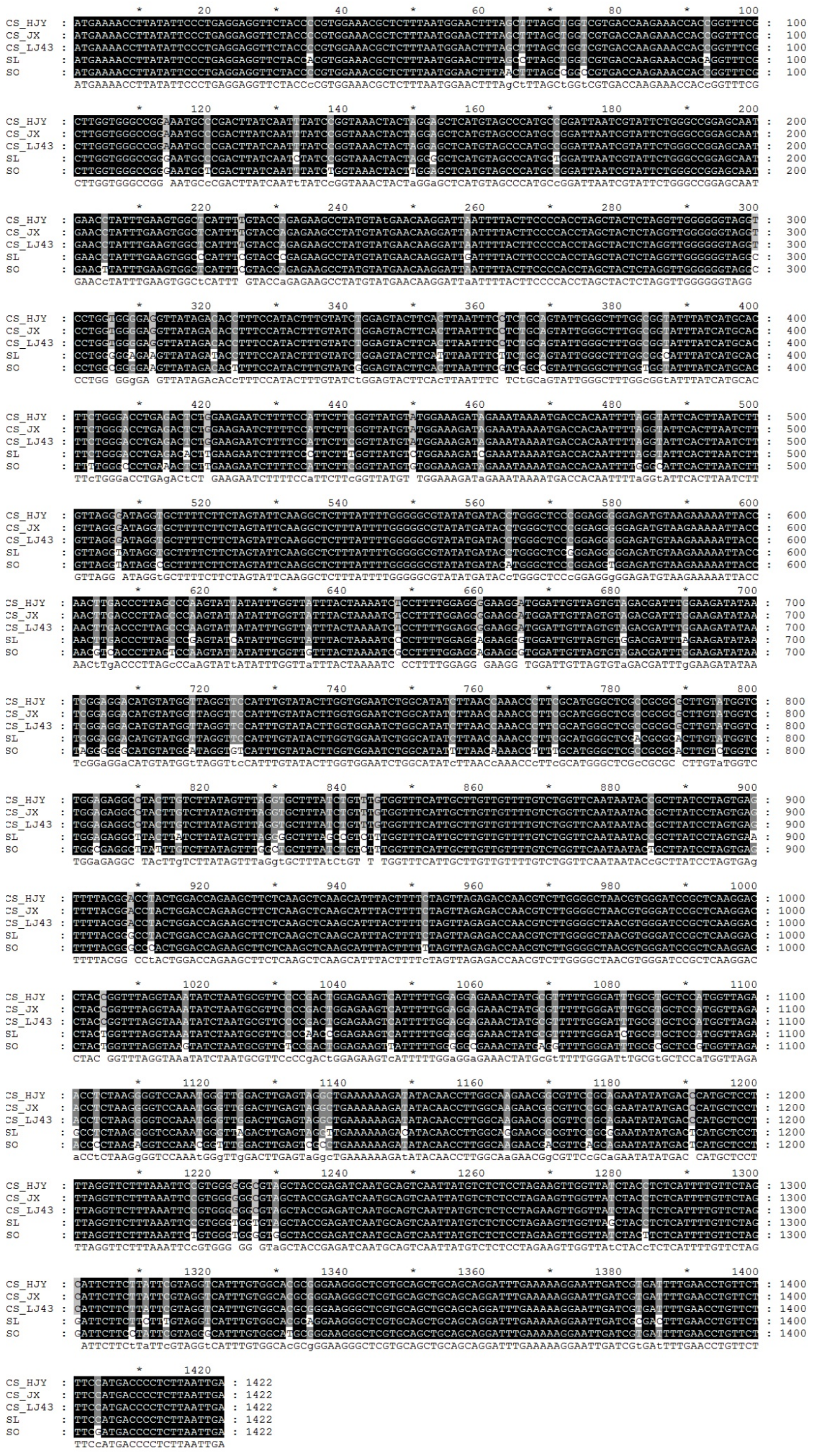

Figure S5. Alignment of psbC cDNA sequences from various plant species. CS_HJY, *Camellia sinensis* cv. Huangjinya; CS_JX, *Camellia sinensis* cv. Jinxuan; CS_LJ43, *Camellia sinensis* cv. Longjing 43; SL, *Solanum lycopersicum*; SO, *Spinacia oleracea*. Data of CS_HJY and CS_JX were detected in the present study and those of CS_LJ43 (GenBank accession No. KF562708.1, position 35705-37126, accessed on 6 Nov.2013), SL (GenBank accession No. DQ347959.1, position 34591-36012, accessed on 13 Jan. 2014) and SO (GenBank accession No. NC_002202.1, position 32516-33937, accessed on 15 Apr. 2009) were obtained by searching from NCBI database.

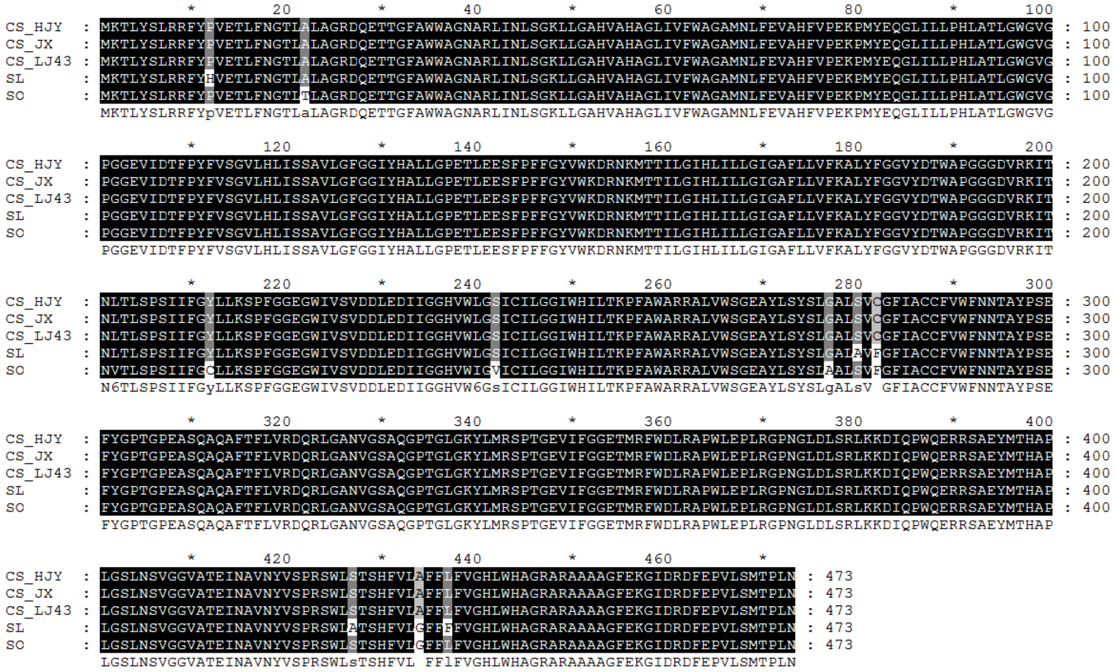

Figure S6. Amino acid alignment of CP43 proteins from various plant species. CS_HJY, *Camellia sinensis* cv. Huangjinya; CS_JX, *Camellia sinensis* cv. Jinxuan; CS_LJ43, *Camellia sinensis* cv. Longjing 43; SL, *Solanum lycopersicum*; SO, *Spinacia oleracea*. Data of CS_HJY and CS_JX were detected in the present study and those of CS_LJ43 (GenBank accession No. KF562708.1, accessed on 06 Nov. 2013), SL (GenBank accession No. DQ347959.1, accessed on 13 Jan. 2014) and SO (GenBank accession No. NC_002202.1, accessed on 15 Apr. 2009) were obtained by searching from NCBI database.
